# The Efficacy of Alendronate Versus Denosumab on Major Osteoporotic Fracture Risk in Elderly Patients With Diabetes Mellitus: A Danish Retrospective Cohort Study

**DOI:** 10.3389/fendo.2021.826997

**Published:** 2022-01-26

**Authors:** Rikke Viggers, Zheer Al-Mashhadi, Jakob Starup-Linde, Peter Vestergaard

**Affiliations:** ^1^ Steno Diabetes Center North Jutland, Department of Endocrinology, Aalborg University Hospital, Aalborg, Denmark; ^2^ Department of Clinical Medicine, Aalborg University, Aalborg, Denmark; ^3^ Steno Diabetes Center Aarhus, Aarhus University Hospital, Aarhus, Denmark; ^4^ Department of Clinical Medicine, Aarhus University, Aarhus, Denmark; ^5^ Department of Endocrinology and Internal Medicine, Aarhus University Hospital, Aarhus, Denmark

**Keywords:** diabetes, fracture, alendronate, denosumab, osteoporosis, bone

## Abstract

**Objective:**

Patients with diabetes mellitus have an increased risk of fractures; however, the underlying mechanism is largely unknown. We aimed to investigate whether the risk of major osteoporotic fractures in diabetes patients differs between subjects initiated with alendronate and denosumab, respectively.

**Methods and Research Design:**

We conducted a retrospective nationwide cohort study through access to all discharge diagnoses (ICD-10 system) from the National Danish Patient Registry along with all redeemed drug prescriptions (ATC classification system) from the Health Service Prescription Registry. We identified all subjects with a diabetes diagnosis between 2000 and 2018 and collected data on the first new prescription of anti-osteoporotic treatment between 2011 and 2018. Exposure was defined as either alendronate or denosumab treatment initiated after diabetes diagnosis. Outcome information was collected by identification of all major osteoporotic fracture (MOF) diagnoses, i.e., hip, spine, forearm, and humerus, from exposure until 2018 or censoring by emigration or death. The risk of fracture was calculated as hazard ratios (HR) using multiply adjusted Cox proportional models with death as a competing risk.

**Results:**

We included 8,745 subjects initiated with either alendronate (n = 8,255) or denosumab (n = 490). The cohort consisted of subjects with a mean age of 73.62 (SD ± 9.27) years, primarily females (69%) and suffering mainly from type 2 diabetes (98.22%) with a median diabetes duration at baseline of 5.45 years (IQR 2.41–9.19). Those in the denosumab group were older (mean 75.60 [SD ± 9.72] versus 73.51 [SD ± 9.23] years), had a higher proportion of women (81% versus 68%, RR 1.18 [95% CI 1.13–1.24], and were more comorbid (mean CCI 2.68 [95% CI 2.47–2.88] versus 1.98 [95% CI 1.93–2.02]) compared to alendronate initiators. In addition, denosumab users had a higher prevalence of previous fractures (64% versus 46%, RR 1.38 [95% CI 1.28–1.48]). The adjusted HR for any MOF after treatment initiation with denosumab was 0.89 (95% CI 0.78–1.02) compared to initiation with alendronate.

**Conclusion:**

The risk of incident MOF among subjects with diabetes was similar between those initially treated with alendronate and denosumab. These findings indicate that the two treatment strategies are equally effective in preventing osteoporotic fractures in subjects with diabetes.

## Introduction

Osteoporosis is an emerging global health problem characterized by microarchitectural deterioration of bone tissue with increased bone fragility and higher fracture risk leading to increased morbidity and mortality (1–3). Diabetes mellitus is a chronic metabolic imbalance associated with increased risk of fractures that cannot be sufficiently predicted by reduced bone mineral density (BMD) (4, 5). In patients with type 1 and type 2 diabetes, the fracture risk may be increased by 7- and 1.3-fold, respectively (4). A current meta-analysis found a relative risk of hip fracture of 4.93 in type 1 diabetes and 1.33 in type 2 diabetes (6). In addition, the relative risk of non-vertebral fractures was found increased by 1.92 and 1.19 in type 1 and type 2 diabetes, respectively (6). Vertebral fractures are often asymptomatic and complex to assess, and thus, data on vertebral fracture risk are sparse (7). Compromised insulin pathways are assumed to cause a deficit in bone structure, reduced osteoblast activity, and a lower number of osteoclasts (8).

Bisphosphonates sufficiently suppress bone resorption by direct inhibition of osteoclast activity, and alendronate, an oral bisphosphonate, is currently the most commonly used treatment of osteoporosis (9). Denosumab is a relatively new treatment of osteoporosis approved as treatment in Denmark in 2010 (10). It is a monoclonal antibody against the receptor activator of nuclear factor-κB ligand (RANKL) which prevents the interaction of RANKL with its receptor, resulting in inhibition of the osteoclast-mediated bone resorption (11, 12).

A more pronounced effect on BMD by denosumab compared to bisphosphonates has been suggested in clinical trials examining postmenopausal women (13–16). In postmenopausal women, it is estimated that alendronate and denosumab increase BMD by 4.7% and 6.0% at the total hip and 6.2% and 9.2% in lumbar spine, respectively (9, 17). In addition, the risk of fractures is reduced by approximately 20%–50% by alendronate and 20%–70% by denosumab; alendronate with the highest protective effect on hip fractures and denosumab on vertebral fractures (17, 18). However, the association between BMD and fracture prediction is not well established (19, 20). Changes in BMD and reduction in fracture risk among users of either alendronate or denosumab are overall similar between subjects with and without diabetes (13, 21, 22). Both alendronate and denosumab treatments are associated with a decreased bone turnover with a more pronounced decrease during denosumab treatment (15). Meta-analyses have shown decreased bone turnover markers in people with diabetes (3, 23, 24). However, bone-specific alkaline phosphatase is reported as normal or increased, suggesting that the bone matrix may become hypermineralized (25). Yet, it is unknown whether a lowering of bone turnover is beneficial and thus alendronate may be superior to denosumab. However, to our knowledge, no studies have investigated potential discrepancies in fracture risk between alendronate and denosumab use in subjects with diabetes.

We aimed to compare the efficacy of alendronate and denosumab treatment on the risk of any new major osteoporotic fracture (MOF), i.e., hip, spine, forearm, and humerus, in subjects with diabetes. We hypothesized that the risk of any MOF was similar after initiation of denosumab compared to alendronate in subjects with diabetes.

## Research Design and Methods

The STROBE statement guideline for reports of observational studies was followed (a STROBE checklist is found in **Supplemental Table S1**) (26).

### Study Design and Setting

We conducted a retrospective nationwide cohort study using information from the Danish national registries. We identified all patients with diabetes between 2000 and 2018 to ensure identification of all individuals with preexisting diabetes and enable an estimation of diabetes duration before exposure. We chose to collect data on exposure of alendronate and denosumab between 2011 and 2018 as denosumab became available as treatment in Denmark in 2010. Outcome information was collected by identifying all fracture-related diagnoses from exposure date until 2018 or censoring date.

### Data Sources

All data were provided and anonymized by Statistics Denmark (*Danmarks Statistik*, project identifier no. 703382) and were obtained through National Danish registries. All Danish citizens are assigned a 10-digit personal identification number which ensures a complete medical history of all contacts to the Danish healthcare system and drug prescriptions for each individual (27–29). The unique person identification number (PIN) has been anonymized and linked to all registries used in this study. All Danish citizens have equal access to full healthcare provided by the Danish National Health Service, which includes free access to hospitals and partial compensation of drug expenses. All authorized Danish research organizations can apply for access to the registries.

Data on diagnoses were obtained from the Danish National Patient Registry (29). The registry covers all contacts to the hospitals on both in- and outpatient bases. The data include all relevant physician-assigned discharge diagnoses on the individual level, coded according to the International Classification of Diseases, Tenth Revision (ICD-10).

Information on drug prescriptions was coded according to the Anatomical Therapeutical Chemical (ATC) classification and recorded from 1996 by the Danish National Health Service Prescription Registry (28, 30). To ensure adequate registration, we collected data from January 1, 2000.

Data on sex and date of birth as well as emigration and death (if applicable) were retrieved from the Danish Civil Registration system, which ensures high-fidelity subject identification with respect to emigration and death (27, 31).

### Study Population

The study population included subjects alive and residing in Denmark with no emigration history on January 1, 2011. We excluded subjects with classified diabetes before January 1, 2000, and individuals of age below 50 years at the index date (initiation of exposure as defined below) (**Supplemental Figure S1**). We chose age 50, as the average age for menopause in Denmark is 51.7 years with a corresponding increase of osteoporosis afterward (32). We excluded subjects treated with other anti-osteoporotic drugs (including alendronate and denosumab) before exposure. Thus, the final cohort consisted of adult individuals with new-onset diabetes between January 1, 2000, and December 31, 2018, who were initiated with either alendronate or denosumab at age ≥ 50 years and after diabetes diagnosis.

### Identification of Diabetes Subjects

Subjects with diabetes mellitus were identified between years 2000 and 2018 either by any ICD-10 code (main or secondary) related to diabetes (E10, E11, E12, E13, E14, G63.2, H28.0, H36.0, M14.2, O24, R73) or by an ATC code of glucose-lowering drugs used in diabetes (A10A or A10B) based on a previously published algorithm (**Supplemental Table S2**) (33–37). The diabetes diagnosis and concordance between actual use and prescription of diabetes-related medications are in general high (38–43). Consequently, all people with diabetes were defined either from a hospital visit or by prescription of glucose-lowering drugs.

In Denmark, all patients with type 1 diabetes will eventually be in contact with the hospital and no other glucose-lowering drugs than insulin were recommended in the study period. Consequently, type 1 diabetes was defined by at least one E10 ICD-10 code (type 1 diabetes) and at least one A10A ATC code (insulins and analogues) and no A10B ATC code (blood glucose-lowering drugs exclusive of insulins); all other individuals with diabetes were classified as type 2 diabetes subjects.

### Exposure: Treatment With Alendronate or Denosumab

All drug prescriptions in Denmark are logged, stored, and linked to the unique PIN. The prescription database includes data on redeemed drugs and corresponding dates, doses, and pack sizes according to the ATC classification system (44).

We defined exposure as a first-ever dispense of either alendronate or denosumab after age 50, after diabetes diagnosis, and after January 1, 2011, using the ATC codes “M05BA04” and “M05BX04”, respectively. The date of the first dispensing of alendronate or denosumab during the study period was set as the index date. We excluded all subjects with any recorded dispensing of other anti-osteoporotic medication (i.e., raloxifene, ipriflavone, strontium ranelate, teriparatide, calcitonin, and other bisphosphonates) before the index date (**Supplemental Table S2**).

We considered subjects as exposed to the initiated drug on the index date, equivalent to the intention-to-treat approach used in randomized controlled trials. To calculate the crude treatment duration, the number of daily doses at the last dispensation date was added to this date, and the date of first drug dispensation was subtracted. The cumulative treatment dose was calculated using a defined daily dose (DDD) of 10 and 0.33 mg for alendronate and denosumab, respectively, based on the World Health Organization Collaborating Centre for Drug Statistics Methodology. Compliance was then assessed using the medication possession ratio (MPR), by dividing the cumulative dose (DDDs) by the treatment duration. MPR was grouped in intervals of a) <0.5, b) 0.5–0.8, and c) ≥0.8, the latter being defined as compliant use. Effective use was defined as the cumulative dose in days if MPR <0.8 and by the crude treatment duration if MPR ≥0.8.

### Outcome: Major Osteoporotic Fractures (MOFs)

Any fracture of the spine, hip, humerus, or forearm was defined as a MOF (45). The primary outcome was any MOF identified by primary or secondary diagnoses during hospitalization by ICD-10 codes (**Supplemental Table S3**) during the follow-up period (between 2011 and 2018). MOF was further categorized into the specific type, i.e., fracture of the spine, hip, humerus, and forearm.

### Identification of Covariates

Covariates at baseline were identified by means of ICD-10 and ATC codes in the period from start date of data collection (January 1, 2000) until the index date (**Supplemental Table S2**). Age at baseline was calculated based on date of birth and date of initiation of treatment.

A history of fracture was identified as any fracture by ICD-10 codes before treatment exposure.

As a proxy for smoking status, we used ICD-10 codes related to lung diseases, of which some were directly and others indirectly associated with tobacco exposure, as well as nicotine poisoning and psychiatric tobacco-related diagnoses (37). In addition, we identified ATC codes corresponding to treatments for tobacco dependence (ever), e.g., nicotine replacement therapy, or initiation of drugs for obstructive airway diseases after the age of 40. Due to potential underestimation, we classified this factor as *heavy smoking*.

We evaluated alcohol consumption by either one relevant ICD-10 or ATC code covering diseases and drugs with direct affiliation to alcohol, e.g., intoxication, alcohol abuse, alcoholic liver disease, alcoholic cardiomyopathy, alcoholic polyneuropathy, alcoholic gastritis, alcohol-induced pancreatitis, or alcohol related psychiatric disorders (37, 46). We classified this factor as *alcohol abuse.*

Obesity was evaluated by ICD-10 codes of obesity or use of anti-obesity pharmaceuticals by ATC codes. Information on chronic and acute pancreatitis was obtained from ICD-10 codes.

Hyper- and hypothyroidism were assessed by either ICD-10 or ATC codes.

Hypertension was defined by any ICD-10 code related to hypertension and/or prescription of any antihypertensive drug. Hypoglycemia was assessed by a related ICD-10 code.

Comorbidity was assessed by use of the Charlson Comorbidity Index (CCI) (47) based on discharge diagnoses registered by ICD-10 codes with a general high accuracy (**Supplemental Table S3**) (48). As alendronate is more or less contraindicated when peptic ulcers or renal impairment is present, we chose to exclude peptic ulcers and nephrological diseases (including those in late diabetes complications) from the index and estimated these as separate variables (**Supplemental Table S2**).

In addition, we identified any prescription of insulins, statins, opioids, glucocorticoids, and anxiolytics by ATC codes up till/at baseline.

Data on socioeconomic status was obtained from Statistics Denmark. We assessed income as the amount of DKK (Danish kroner) from the year preceding the year of index and adjusted for inflation to a 2018 level using the consumer price index from Statistics Denmark. Lastly, we converted the income to euro € at a rate of 1 € = 7.467 DKK (exchange rate December 2018) and grouped into quintiles for analysis.

Marital status was available through the Danish Civil Registration System and assessed from the year prior to the year of index. It was defined and grouped according to the classification from Statistics Denmark: married, divorced, widowed, or unmarried.

### Statistical Analysis

The study period was defined as time from exposure initiation, i.e., initiation of treatment with either alendronate or denosumab (index date), until the date of a MOF outcome, death, emigration, or December 31, 2018, whichever occurred first.

Descriptive statistics are presented as numbers (n) and percentages (%), means and standard deviations (SD), or medians and interquartile ranges (IQR). Unpaired t-tests and chi-square tests were used to compare continuous and dichotomous variables across exposure groups. Differences in exposure groups are presented as mean differences or risk ratios (RR) with 95% confidence intervals (CI).

We plotted exposure-specific cumulative incidence curves for any first MOF, considering death as a competing risk by performing a competing risk regression analysis fitted by Fine and Gray’s proportional sub-distribution hazard models (49) with death as a competitive event and alendronate exposure as comparator. Crude and adjusted hazard rate ratios (HR) with 95% CI were estimated for each outcome. We examined the assumption of proportionality by graphical log-log plots, and no violation was identified. With respect to multicollinearity, we performed a multiple adjustment. Interactions were evaluated and found significant between age and a history of fracture with no difference in results after incorporating the main effects and interaction effect in our primary analysis. Thus, we made a subgroup analysis stratified by fracture history, age (< and ≥75 years), and sex.

We performed several sensitivity analyses. Firstly, we excluded all subjects with type 1 diabetes from the cohort. In addition, we included censoring at any discontinuation of treatment due to a switch from the initial treatment to another anti-osteoporotic drug, i.e., per-protocol approach. As denosumab has a faster clearance than alendronate (50), we further stratified this sensitivity analysis (censoring at switch in treatment) on effective use. Moreover, we performed a sensitivity analysis including censoring at switch in treatment and at discontinuation (last date of drug prescription with addition of amount of DDD in the dispense) and made a modified analysis by censoring 1 year after discontinuation of treatment. Furthermore, we performed a sensitivity analysis only on subjects with high adherence, i.e., MPR ≥0.8. As glucocorticoids are known to impact on bone quality, we performed a sensitivity analysis only including those who used glucocorticoids up till/at baseline.

Lastly, we identified and displaced subjects with a switch in treatment from alendronate to denosumab within 6 months to the denosumab group.

All analyses were conducted in STATA 16.1 (StataCorp, College Station, Texas, US).

### Resource Availability

Data were available and anonymized by Statistics Denmark. All authorized Danish research organizations can apply for access.

Approval by the ethics committee is not required for epidemiological studies in Denmark. We had no access to personally identifiable information and the registries are subject to control by the Danish Data Protection Agency.

## Results

### Baseline Characteristics

We identified 8,745 elderly subjects with new onset diabetes mellitus with initiated anti-osteoporotic treatment of either alendronate (n = 8,255) or denosumab (n = 490) after diabetes diagnosis and without any history of anti-osteoporotic treatment.

**Table 1** shows baseline characteristics of subjects initiated with alendronate and denosumab. In general, the cohort consisted of elderly subjects with mean ( ± SD) age 73.62 ( ± 9.27) years suffering mainly from type 2 diabetes (98.22% [95% CI 97.92–98.48]) with a median (IQR) diabetes duration at baseline of 5.45 years (2.41–9.19).

**Table 1:** Baseline characteristics of subjects initiated with alendronate and denosumab after diabetes diagnosis in Denmark from 2011 to 2018.

	All subjects n = 8,745	Alendronate n = 8,255	Denosumab n = 490
**Age (years),** mean ± SD	73.62 (9.27)	73.51 (9.23)	75.60 (9.72)
**Age category (years)**, n (%)			
50–59	755 (9)	720 (9)	35 (7)
60–69	2,196 (25)	2,100 (25)	96 (20)
70–79	3,481 (40)	3,293 (40)	188 (38)
≥80	2,313 (26)	2,142 (26)	171 (35)
**Sex,** n (%)			
Female	6,043 (69)	5,647 (68)	396 (81)
Male	2,702 (31)	2,608 (32)	94 (19)
**Type 2 diabetes**, n (%)	8,589 (98)	8,114 (98)	475 (97)
**Diabetes duration in years,** median (IQR)	5.45 (2.41-9.19)	5.43 (2.41-9.18)	5.57 (2.34-9.52)
**History of any fracture**, n (%)	4,141 (47)	3,828 (46)	313 (64)
**CCI,** mean ± SD	1.81 (1.89)	1.78 (1.88)	2.26 (2.07)
**CCI categories,** n (%)			
0	2,609 (30)	2,491 (30)	118 (24)
1	1,963 (22)	1,879 (23)	84 (17)
≥2	4,173 (48)	3,885 (47)	288 (59)
**Peptic ulcer,** n (%)	507 (6)	455 (6)	52 (11)
**Renal impairment,** n (%)	693 (8)	615 (7)	78 (16)
**Income,** € in thousands, median (IQR)	26.13 (19.85-32.54)	26.11 (19.85-32.58)	26.44 (19.92-31.53)
1^st^ quintile, n (%)	1,749 (20)	1,645 (20)	104 (21)
2^nd^ quintile, n (%)	1,749 (20)	1,677 (20)	72 (15)
3^rd^ quintile, n (%)	1,749 (20)	1,637 (20)	112 (23)
4^th^ quintile, n (%)	1,749 (20)	1,637 (20)	112 (23)
5^th^ quintile, n (%)	1,749 (20)	1,659 (20)	90 (18)
**Marital status,** n (%)			
Married	4,241 (49)	4,015 (49)	226 (56)
Divorced	1,358 (16)	1,280 (16)	78 (16)
Unmarried	606 (6.93)	574 (7)	32 (7)
Widowed	2,534 (29)	2,380 (29)	154 (31)
Unknown	6 (0)	6 (0)	0 (0)
**Heavy smoking,** n (%)	3,116 (36)	2,927 (35)	189 (39)
**Alcohol abuse,** n (%)	747 (9)	708 (9)	39 (8)
**Obesity,** n (%)	1,543 (18)	1,457 (18)	86 (18)
**Pancreatitis,** n (%)	298 (3)	283 (3)	15 (3)
**Hyperthyroidism,** n (%)	279 (3)	254 (3)	25 (5)
**Hypothyroidism,** n (%)	629 (7)	589 (7)	40 (8)
**Glucocorticoid use,** n (%)	5,027 (57)	4,757 (58)	270 (55)
**Statin use**, n (%)	6,791 (78)	6,424 (78)	367 (75)
**Insulin use**, n (%)	1,654 (19)	1,543 (19)	111 (23)
**Hypoglycemia**, % ± SD	193 (2)	177 (2)	16 (3)
**Hypertension**, n (%)	7,996 (91)	7,543 (91)	453 (92)
**Opioid use**, n (%)	6,755 (77)	6,342 (77)	413 (84)
**Anxiolytics**, n (%)	7,741 (89)	7,286 (88)	455 (93)
**Initiation year**, n (%)			
2011	1,098 (13)	1,018 (12)	80 (16)
2012	1,052 (12)	989 (12)	63 (13)
2013	1,076 (12)	1,018 (12)	58 (12)
2014	1,100 (13)	1,044 (13)	56 (11)
2015	1,072 (13)	1,021 (12)	51 (10)
2016	1,113 (13)	1,050 (13)	63 (13)
2017	1,155 (13)	1,099 (13)	56 (11)
2018	1,079 (12)	1,016 (12)	63 (13)

All characteristics were evaluated in the time from 2000 until the index date (exposure start). Data are presented as numbers (n, %), mean with SD or median with IQR.

Subjects initiated with denosumab were older, mean ( ± SD) 75.60 ( ± 9.72) versus 73.51 ( ± 9.23) years (p < 0.001), had a higher proportion of women in the cohort (81% versus 68%, RR 1.18 [95% CI 1.13–1.24), and were more comorbid (mean CCI 2.26 [96% CI 2.07–2.44] versus 1.78 [95% CI 1.74–1.82]) compared to alendronate initiators. In addition, denosumab exposed individuals had a higher prevalence of previous fractures (64% versus 46%, RR 1.38 [95% CI 1.28–1.48]), a higher proportion of renal impairment (11% versus 6%, RR 1.93 [95% CI 1.47–2.53]), and a higher prevalence of peptic ulcers (16% versus 7%, RR 2.14 [95% CI 1.72–2.66]). There was no difference in marital status or income, either on total income or within each quintile, between subjects initiated with alendronate and denosumab. A higher proportion of those treated with denosumab had hyperthyroidism (5.10% versus 3.08%, RR 1.66 [95% CI 1.11–2.48]), and they were more frequently users of insulin (22.65% versus 18.69%, RR 1.21 [95% CI 1.02–1.44]), opioids (84.29% versus 76.83%, RR 1.10 [95% CI 1.05–1.14]), and anxiolytics (92.86% versus 88.26%, RR 1.05 [95% CI 1.03–1.08]) compared to the alendronate group. There was no significant difference in smoking, alcohol, obesity, pancreatitis, hypothyroidism, glucocorticoid use, statin use, hypoglycemia, or hypertension between exposure groups.

### Risk of Major Osteoporotic Fractures

Median (IQR) follow-up time was 2.67 (1.17–4.62) years among alendronate initiators and 2.36 (0.95–4.53) years among denosumab initiators. Deaths during follow-up were more frequent in the denosumab group (27% versus 34%, RR 1.29 [95% CI 1.14–1.47]).

Median treatment duration in days (defined by cumulative DDD) of alendronate and denosumab was 560 days (IQR 182–1,218) and 727 days (IQR 363–1,455), respectively.

**Figure 1:**
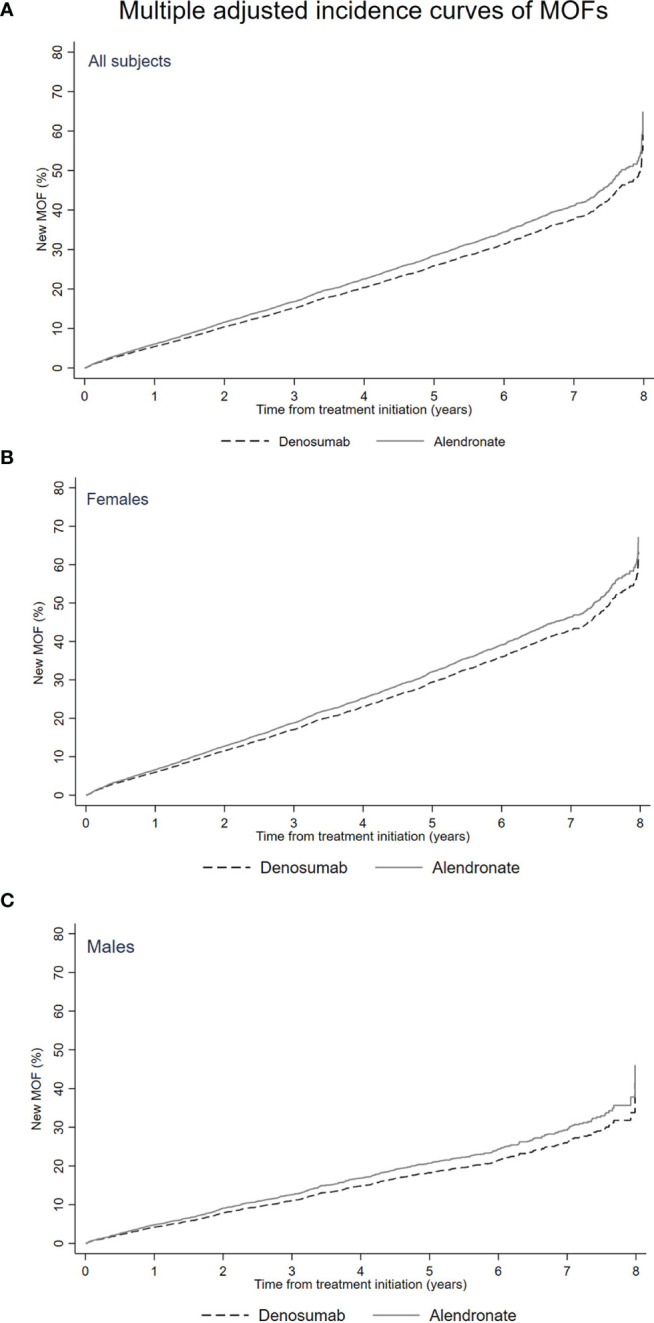
Multiple adjusted cumulative incidence curve of any first MOF following initiation of alendronate or denosumab (primary analysis). **(A)** All subjects. **(B)** Females. **(C)** Males.

**Table 2** and **Figure 1** present risk of MOFs during the study period. A new MOF occurred in 49% (n = 238) and 39% (n = 3,256) of denosumab and alendronate initiators, respectively. Crude HR for any MOF during the study period among initiators of denosumab was 1.26 (95% CI 1.10–1.44) with initiators of alendronate as reference. The risk was entirely attenuated in the fully adjusted model (HR 0.89 [95% CI 0.89–1.02]). Stratification by age (75-year cutoff), sex, and a history of any fracture did not change the risk of any MOF significantly (**Table 2**).

**Table 2:** Risk of MOF and stratification by age, sex, history of any fracture, and MOF type.

	Exposure	MOF, n (%)	Hazard ratios (HR) and 95% CI			
			Crude	Adjusted 1^a^	Adjusted 2^b^	Adjusted 3^c^
**Overall**	Denosumab	238 (49)	1.26 (1.10–1.44)	1.17 (1.03–1.34)	0.92 (0.80–1.05)	0.89 (0.78–1.02)
	Alendronate	3,256 (39)	1 (reference)	1 (reference)	1 (reference)	1 (reference)
**Age category**						
<75 years	Denosumab	88 (37)	1.18 (0.95–1.47)	1.14 (0.92–1.42)	0.93 (0.81–1.06)	0.80 (0.64–1.00)
	Alendronate	1,504 (46)	1 (reference)	1 (reference)	1 (reference)	1 (reference)
≥75 years	Denosumab	150 (63)	1.23 (1.04–1.46)	1.20 (1.02–1.42)	0.98 (0.83–1.16)	1.97 (0.82–1.16)
	Alendronate	1,752 (54)	1 (reference)	1 (reference)	1 (reference)	1 (reference)
**Sex**						
Female	Denosumab	203 (85)	1.19 (1.03–1.38)	1.17 (1.01–1.35)	0.93 (0.80–1.08)	0.90 (0.77–1.04)
	Alendronate	2,416 (74)	1 (reference)	1 (reference)	1 (reference)	1 (reference)
Male	Denosumab	35 (15)	1.29 (0.91–1.82)	1.20 (0.85–1.26)	0.85 (0.61–1.19)	0.86 (0.63–1.26)
	Alendronate	840 (26)	1 (reference)	1 (reference)	1 (reference)	1 (reference)
**History of any fracture**						
Yes	Denosumab	218 (92)	0.90 (0.78–1.05)	0.89 (0.77–1.03)	–	0.87 (0.75–1.01)
	Alendronate	2,863 (88)	1 (reference)	1 (reference)	–	1 (reference)
No	Denosumab	20 (8)	1.23 (0.78–1.94)	1.12 (0.72–1.75)	–	1.13 (0.72–1.77)
	Alendronate	393 (12)	1 (reference)	1 (reference)	–	1 (reference)
**Type of first MOF**						
Spine	Denosumab	45 (19)	1.13 (0.84–1.53)	1.14 (0.84–1.53)	0.90 (0.67–1.21)	0.82 (0.59–1.15)
	Alendronate	684 (21)	1 (reference)	1 (reference)	1 (reference)	1 (reference)
Hip	Denosumab	98 (41)	1.31 (1.06–1.62)	1.20 (0.97–1.48)	1.07 (0.87–1.32)	0.93 (0.75–1.16)
	Alendronate	1,289 (40)	1 (reference)	1 (reference)	1 (reference)	1 (reference)
Humerus	Denosumab	33 (14)	1.31 (0.92–1.87)	1.20 (0.97–1.48)	0.95 (0.77–1.17)	0.91 (0.63–1.29)
	Alendronate	434 (13)	1 (reference)	1 (reference)	1 (reference)	1 (reference)
Forearm	Denosumab	62 (26)	1.25 (0.97–1.62)	1.13 (0.87–1.46)	0.87 (0.67–1.13)	0.87 (0.66–1.14)
	Alendronate	849 (26)	1 (reference)	1 (reference)	1 (reference)	1 (reference)

MOF, n (%) represents number and % of MOFs in each category by exposure. Adjusted HRs (95% CIs) with alendronate exposure as reference with exclusion of stratified category in adjusted analyses.

a Adjusted for sex and age.

b Adjusted for sex, age, history of fracture.

c Multiple adjustment for sex, age, history of fractures, diabetes duration, insulin, hypoglycemia, anxiolytics, statin, opioid, smoking, alcohol, glucocorticoid, pancreatitis, hypo- and hyperthyroidism, peptic ulcer, renal impairment, CCI, income, and marital status.

Hip fractures were the most prevalent type of MOF in both exposure groups followed by fractures of the forearm, spine, and humerus (**Table 2**). The risk of hip fracture as first MOF was similar between groups (adjusted HR 0.93 [95% CI 0.75–1.16]).

### Sensitivity Analysis

**Table 3** presents data from 6 sensitivity analyses.

**Table 3:** Risk of MOF in sensitivity analyses.

	Exposure	MOF, n (%)	Hazard ratios (HR) and 95% CI			
			Crude	Adjusted 1^a^	Adjusted 2^b^	Adjusted 3^c^
**1,** Type 2 diabetes	Denosumab	232 (49)	1.25 (1.10–1.44)	1.17 (1.02–1.34)	0.92 (0.80–1.05)	0.89 (0.78–1.02)
	Alendronate	3,204 (39)	1 (reference)	1 (reference)	1 (reference)	1 (reference)
**2,** MPR ≥ 0.8	Denosumab	222 (49)	1.23 (1.07–1.41)	1.17 (1.02–1.34)	0.90 (0.79–1.04)	0.89 (0.77–1.02)
	Alendronate	2,777 (40)	1 (reference)	1 (reference)	1 (reference)	1 (reference)
**3,** Censor at switch	Denosumab	209 (43)	1.33 (1.15–1.53)	1.24 (1.08–1.43)	0.92 (0.80–1.07)	0.89 (0.76–1.03)
	Alendronate	2,683 (33)	1 (reference)	1 (reference)	1 (reference)	1 (reference)
**4,** Censor switch and discontinuation	Denosumab	209 (43)	1.42 (1.20–1.68)	1.34 (1.13–1.59)	1.02 (0.86–1.21)	0.97 (0.82–1.16)
	Alendronate	2,683 (33)	1 (reference)	1 (reference)	1 (reference)	1 (reference)
**5**, Censor 1 year discontinuation	Denosumab	209 (43)	1.34 (1.15–1.56)	1.26 (1.08–1.46)	0.92 (0.81–1.10)	0.91 (0.78–1.06)
	Alendronate	2,683 (33)	1 (reference)	1 (reference)	1 (reference)	1 (reference)
**6,** Switch < 6 months	Denosumab	271 (47)	1.22 (1.08–1.39)	1.15 (1.02–1.30)	0.92 (0.81–1.04)	0.89 (0.79–1.01)
	Alendronate	3,223 (39)	1 (reference)	1 (reference)	1 (reference)	1 (reference)
**7,** Glucocorticoid users	Denosumab	123 (46)	1.44	1.32 (1.09–1.59)	1.01 (0.84–1.22)	0.99 (0.83–1.20)
	Alendronate	1,602 (34)	1 (reference)	1 (reference)	1 (reference)	1 (reference)

Risk of MOF in 5 sensitivity analyses. 1, Only including subjects with type 2 diabetes. 2, Only including subjects with high compliance/drug adherence (MPR ≥ 0.8). 3, Censoring at switch in anti-osteoporotic treatment. 4, Censoring at switch in or discontinuation of anti-osteoporotic treatment. 5, Censoring at switch in anti-osteoporotic treatment and 1 year after discontinuation. 6, Subjects displaced to denosumab users if a switch from alendronate to denosumab was set within 6 months of treatment. 7, Only including users of glucocorticoid up till/at baseline. MOF, n (%) represents numbers and % of MOFs in each category by exposure. Adjusted HRs (95% CIs) with alendronate exposure as reference.

a Adjusted for sex and age.

b Adjusted for sex, age, history of fracture.

c Multiple adjustment for sex, age, history of fractures, diabetes duration, insulin, hypoglycemia, anxiolytics, statin, opioid, smoking, alcohol, glucocorticoid, pancreatitis, hypo- and hyperthyroidism, peptic ulcer, renal impairment, CCI, income, and marital status.

The risk of any MOF did not change after excluding subjects with type 1 diabetes from the cohort (adjusted HR 0.89 [95% CI 0.78–1.02)]) or those with low adherence (MPR <0.8 (adjusted HR 0.89 [0.77–1.02)]). Neither were there any difference in the risk of MOF between alendronate and denosumab initiators when only including subjects with use of glucocorticoids up till/at baseline.

In total, 4,078 (47%) subjects discontinued the original treatment before end of follow-up (**Supplemental Table S5**). Of these, 3,484 subjects discontinued without any prescription of other anti-osteoporotic treatment; 149 from the denosumab group and 3,284 from the alendronate group, corresponding to 30% and 42%, respectively. Of those who discontinued, 445 replaced the original treatment with another anti-osteoporotic treatment before end of follow-up; 274 subjects switched from alendronate to denosumab, 7 subjects switched from denosumab to alendronate and 165 subjects switched to a third anti-osteoporotic drug of which all were alendronate initiators (1 subject switched from alendronate to denosumab and lastly to a third drug). Baseline characteristics of subjects discontinuing the original treatment did not differ from the original cohort (**Supplemental Table S5**). The numbers and risks of any MOF did not change after censoring at switch in treatment, at discontinuation, or 1 year after discontinuation. After displacing subjects with a switch in treatment from alendronate to denosumab within 6 months to the denosumab exposure group (n = 81), the risk of MOF did not change.

Stratification by type of MOF and by effective use did not reveal any differences in the risk of MOF between exposure groups.

## Discussion

This cohort study examined the risk of any first MOF among subjects with diabetes after initiation with anti-osteoporotic treatments of denosumab or alendronate. The risk of any MOF after treatment initiation between users of alendronate and denosumab was similar during the follow-up period, although the estimates moved toward a protective effect of denosumab after multiple adjustments. Hip fractures were the most frequent type of MOF in both alendronate and denosumab initiators without difference in risk.

In clinical practice, increased BMD is expected as an adequate response to therapy and results in a significant reduction in fracture risk (51). Long-term studies suggest larger BMD gain after denosumab use compared to alendronate with no difference in safety and adverse events (15, 16). In addition, a large cohort study found similar fracture risks between users of denosumab and alendronate (52). However, these studies did not include analyses on subjects with diabetes. As BMD is often inappropriately high in patients with type 2 diabetes (4), a proper response to anti-osteoporotic therapy in patients with diabetes is restricted to assessment of fracture risk, an endpoint hard to evaluate in clinical trials as it requires long-term follow-up. Our study supports the overall hypothesis of no difference between alendronate and denosumab on fracture risk in diabetes. However, to our knowledge, no other studies have examined the risk of fractures between denosumab and alendronate users in this setting.

Several studies on diabetic animals have provided solid evidence of a reduction in fracture risk with anti-osteoporotic treatments (53, 54), based on their ability to increase BMD and bone strength. Although diabetes is characterized by low bone turnover, further reduction of bone turnover with antiresorptive therapies does not seem to negatively affect the potential to prevent fractures (55). A systematic review from 2018 identified 9 studies and found no differences in the efficacy of anti-osteoporotic medications on fracture risk and BMD changes in patients with diabetes; however, no eligible studies were identified to evaluate denosumab (56). In humans, available data are scarce and mainly obtained from *post hoc* analyses of large osteoporosis RCTs. In a *post hoc* analysis, 3 years of alendronate treatment increased BMD at all sites compared to a placebo group, and the increase was similar in subjects without incident diabetes (57). In addition, treatment with denosumab increased BMD at all sites irrespective of the presence of diabetes and reduced the risk for new vertebral fractures; however, the study revealed a higher incidence of non-vertebral fractures (mostly forearm and ribs) in subjects with diabetes (22). We did not find a higher risk of forearm fractures among initiators of denosumab compared to alendronate.

The effect of transition from alendronate to denosumab or risedronate has been investigated previously. A recent observational study investigated switching from bisphosphonates to denosumab but did not find any BMD improvement after 6 months of denosumab treatment in patients with type 2 diabetes with prior bisphosphonate use (58). An RCT comparing women with suboptimal adherence to alendronate therapy found a higher BMD increase and reduced bone turnover 12 months after a switch to denosumab compared to risedronate (59). Another RCT found that discontinuation of alendronate did not affect fracture risk after 5 years without treatment (60). As alendronate has a long half-life, a potential benefit from a switch may, in part, be the long-lasting or an additive effect of alendronate. In our sensitivity analysis, no change in risk of any MOF was found after censoring those with a switch in treatment or discontinuation. In addition, we did not observe any differences after stratification by effective use or after exclusion of those with low adherence to the initiated treatment. Lastly, we did not observe a difference in the risk of any MOF after displacing subjects with a switch in treatment from alendronate to denosumab within 6 months of treatment.

Falls is another possible cause of fractures in patients with diabetes (61). It has been suggested that denosumab improves muscle mass and strength, and thus, may have the potential to reduce fall risk, which may in turn lower the risk of fractures (62). However, the aforementioned higher incidence of forearm and rib fractures in denosumab uses may also indicate a higher fall rate (22). On the other hand, current research suggests a possible protective effect of alendronate on the risk of developing type 2 diabetes as well as reducing insulin consumption and improving insulin sensitivity in prediabetes (37, 63–67); factors which could decrease the risk of late diabetes complications and, thereby, fracture risk (68).

One notable strength of the current study is the utility of the Danish National Registers based on the unique personal identification number assigned to all Danish citizens with high quality and validity (27, 29, 69, 70). Furthermore, the identification of people with diabetes in Denmark was nationwide without any selection bias. Another strength was the ability to include a high number of potential confounders. It is highly possible that denosumab initiation is preferred in older patients and in those with peptic ulcers and renal impairment. However, we were able to adjust for these covariates by means of ICD-10 codes, and the risk of MOF did not change when only including subjects with high adherence to the drug or after displacement of switchers from alendronate to denosumab within 6 months of treatment.

Though few adverse events have been reported after initiation of alendronate (71), these events are rarely reported after initiation of denosumab (17). This may lead to differences in treatment indication, or consequently, a switch in treatment from alendronate to denosumab as seen in our cohort. However, we would expect most changes in treatment due to adverse events to occur within 6 months after treatment initiation. In addition, we compare a newer agent with an established treatment and cannot dismiss the possibility of residual confounding. For example, we did not have access to laboratory results, e.g., glycemic control, BMI, or BMD measurements, all of which may influence on bone microarchitecture and fracture risk (72). Furthermore, some fractures, especially spine fractures, may go undetected, and this may have led to an underreporting of MOFs in our analysis. However, underreporting of vertebral fractures is expected to be similar between the two groups; therefore, we do not expect this to affect the results in either direction. In addition, the median follow-up time was just above 2 years and may as well underestimate the evaluation of fracture risk. A higher proportion of denosumab users had renal impairment and peptic ulcers compared to alendronate initiators and could potentially have a lower BMD at treatment initiation. Although we were able to adjust for two of these factors, it is possible that these are incompletely measured by ICD-10 codes, allowing confounding by indication. Those initiated with alendronate were in general less comorbid than those initiated with denosumab, while a higher proportion of deaths occurred in the denosumab group, which may lead to a healthy survivor bias. We chose to perform a competitive regression analysis as well as adjusting for a highly validated comorbidity index to minimize this bias (48). Lastly, as we excluded subjects with diabetes before January 1, 2000, and individuals of age below 50 years at index date (year 2011 as the earliest), naïve type 1 diabetes patients included in this cohort were older than a typical type 1 diabetes patient underestimating the proportion of subjects with type 1 diabetes compared to type 2 diabetes. As patients with type 1 diabetes have a higher fracture risk compared to type 2 diabetes, this might underestimate the fracture rate (4). However, excluding individuals with type 1 diabetes from the cohort did not affect our results.

Although the Danish registries contain a wide range of validated information, we did not have access to over-the-counter-medicine, e.g., vitamin D supplementation, or information of lifestyle factors such as diet and exercise. In addition, the registries did not include data on smoking habits and alcohol consumptions; however, we estimated some of these baseline characteristics using ICD-10 and ATC codes as proxies. Consequently, we only obtained these covariates from subjects with already developed concomitant disease or with prescribed medical therapy.

In conclusion, subjects with diabetes initiated with denosumab have a similar risk of a new major osteoporotic fracture as subjects initiated with alendronate. The risk was not associated with sex, age, or a history of fractures. Alendronate appears to be the first choice in treatment of osteoporosis irrespectively of the presence of diabetes. To our knowledge, there are no specific treatment recommendations available for osteoporosis in the presence of diabetes, and it is our hope that the current findings may encourage attention to the cross-link between bone health and diabetes. We propose future research to prospectively evaluate anti-osteoporotic treatments in patients with diabetes, e.g., by basic metabolic research, acute intervention trials and randomized controlled trials including head-to-head comparison of the effects of denosumab and alendronate on bone indices in subjects with diabetes. As BMD is an insufficient measure of fracture risk, more data are needed to clarify whether there are any differences in the efficacy of anti-osteoporotic drugs on other bone indices and fracture risk in subjects with diabetes.

## Data Availability Statement

The datasets presented in this article are not readily available because only authorized Danish research organizations can apply for access. Requests to access the datasets should be directed to p.vestergaard@rn.dk.

## Author Contributions

All authors contributed to the article according to the ICJME requirements for coauthorship. All authors had full access to all data used in the study, critically revised the paper for intellectual content, and approved submitted versions and the final version of the paper. RV and PV designed the study. RV performed the data management and statistical analyses with assistance from all coauthors. RV and PV interpreted the data. RV wrote the paper. ZA-M and JS-L made ongoing critical revisions of data management, design, and data interpretation and reviewed the manuscript. All authors contributed to the article and approved the submitted version.

## Funding

This work was supported by a Steno Collaborative grant, Novo Nordisk Foundation, Denmark (Grant no. NNF18OC0052064).

## Conflict of Interest

The authors declare that the research was conducted in the absence of any commercial or financial relationships that could be construed as a potential conflict of interest.

## Publisher’s Note

All claims expressed in this article are solely those of the authors and do not necessarily represent those of their affiliated organizations, or those of the publisher, the editors and the reviewers. Any product that may be evaluated in this article, or claim that may be made by its manufacturer, is not guaranteed or endorsed by the publisher.
